# Characteristics of Air Pollutants Emission and Its Impacts on Public Health of Chengdu, Western China

**DOI:** 10.3390/ijerph192416852

**Published:** 2022-12-15

**Authors:** Ju Wang, Juan Li, Xinlong Li, Chunsheng Fang

**Affiliations:** 1 College of New Energy and Environment, Jilin University, Changchun 130012, China; 2 Key Laboratory of Groundwater Resources and Environment, Ministry of Education, Jilin University, Changchun 130012, China; 3 Jilin Province Key Laboratory of Water Resources and Environment, Jilin University, Changchun 130012, China

**Keywords:** PM_2.5_, Chengdu, emission inventory, WRF-CMAQ, burden of mortality

## Abstract

Pollution caused by PM_2.5_ and O_3_ are common environmental problems which can easily affect human health. Chengdu is a major central city in Western China, and there is little research on the regional emissions and health effects of air pollution in Chengdu. According to the Multi-resolution Emissions Inventory of the Chinese Model, 2017 (MEIC v1.3), this study compiled the air pollutant emission inventory of Chengdu. The results show that the pollutant emission of Chengdu is generally higher in winter than in summer. The southeast area of Chengdu is the key area where emissions of residential and industrial sectors are dominant. Through air quality simulation with a Weather Research and Forecasting model, coupled with the Community Multiscale Air Quality (WRF-CMAQ), the health effects of PM_2.5_ and O_3_ in winter and summer in Chengdu of 2017 were investigated. The primary pollutant in winter is PM_2.5_ and O_3_ in summer. PM_2.5_ pollution accounted for 351 deaths in January and July 2017, and O_3_ pollution accounted for 328 deaths in the same period. There were 276 deaths in rural areas and 413 in urban areas. In January and July 2017, the health economic loss caused by PM_2.5_ accounted for 0.0974% of the gross regional product (GDP) of Chengdu in 2017, and the health economic loss caused by O_3_ accounted for 0.0910%.

## 1. Introduction

Air pollution is one of the universal environmental problems in the world. The environmental pollution caused by the concentration of fine particulate matter (PM_2.5_) and ozone (O_3_) exceeding the air quality standard has attracted people’s attention [1,2,3,4,5]. Fine particulate matter in the air with small particle size can be suspended in the air for a long time; its composition is complex, containing sulfate, nitrate and other chemical components, as well as toxic trace elements [6,7]. PM_2.5_ has negative impacts on health by triggering inflammatory, mutagenic/genotoxic, and intracellular oxidative stress responses [8]. Atmospheric ozone pollution is due to anthropogenic emissions of pollutants of many precursors including VOCs and NO_x_ through photochemical reactions [9]. There is a positive and independent association between long-term O_3_ exposure and cause-specific cardiovascular mortality [10]. Nearly 18% of respiratory mortality in Shanghai from 2013 to 2019 was attributed to O_3_ exposure [11].

In order to clarify the impact of air pollution on human health, many scholars have conducted research from the perspective of health exposure risk [12,13,14,15,16]. Hu et al. [17] estimated the PM_2.5_ premature mortality amount was about 3.15 million/year in 2010 globally, and China is the leading country, with about 1.33 million/year. Ding, D et al. [18] indicated that the total mortality of PM_2.5_ in China in 2013 was estimated to be 1.389 million (95%CI: 10.05 million, 1.631 million), and in 2017 was estimated to be 1.102 million (95%CI: 0.775 million, 1.337 million), the health burden of PM_2.5_ pollution in China is much higher than the other developed countries. Studies show that respiratory mortality and non-accidental mortality increased by 1.24% (95%CI: 0.29%, 2.22%) and 0.36% (95%CI: 0.10%, 0.63%) with the increase of PM_2.5_ by 10 μg/m^3^. For every 10 μg/m^3^ increase in O_3_, respiratory mortality and non-accidental mortality increased by 2.22% (95%CI: 0.56%, 3.90%) and 0.05% (95%CI: 0.42%, 0.53%), respectively [19]. In 2030, without controlling policies, PM_2.5_ pollution could lead to a loss of 2.0% of gross regional product (GDP), and O_3_ pollution could lead to a loss of 0.09% of GDP [20].

In addition to being a significant metropolis in the center of western China, Chengdu also serves as the business, financial, and transportation hub for southwest China. There is, however, limited research on the emission of pollutants and their consequences on health in various locations within Chengdu.

In this paper, a Weather Research and Forecasting model coupled with the Community Multiscale Air Quality (WRF-CMAQ) is used to simulate the air quality of Chengdu in January and July 2017, to consummate the monitoring value and obtain complete air quality data. Based on this, the emission of pollutants in winter and summer and the health and economic losses caused by PM_2.5_ and O_3_ in Chengdu were studied. The study provides insights into the development of air quality control strategies in Chengdu.

## 2. Data and Methods

### 2.1. WRF-CMAQ Model and the Study Area

The WRF-CMAQ model is used to simulate the air quality of Chengdu in January (winter) and July (summer) in 2017. The atmospheric pollutants concentration data of environmental quality automatic monitoring station of Chengdu from China’s environmental monitoring station of national urban air quality real-time publishing platform (http://beijingair.sinaapp.com/) (accessed on 5 July 2022) and the meteorological data of Shuangliu District (SL) meteorological station from China meteorological data service center (http://data.cma.cn/) (accessed on 10 July 2022) were collected for WRF-CMAQ model simulation test and the analysis of research. The Community Multiscale Air Quality model (CMAQ v5.3.2) (https://www.cmascenter.org/cmaq/) (accessed on 3 July 2022) model is used in this study.

**Figure 1:**
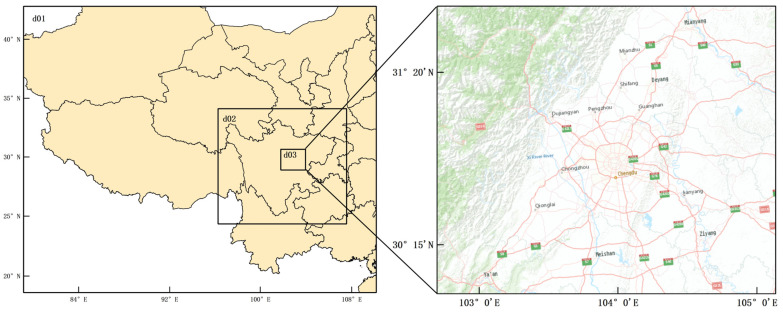
Three-level nested modeling domains.

Figure 1 shows the nested domain used in this study. Domain 1 covers most of western China, including Sichuan Province, Guizhou Province, Chongqing Municipality, Yunnan Province, Tibet Autonomous Region, Qinghai Province, Gansu Province, Shaanxi Province, with a mesh resolution of 27 km × 27 km; Domain 2 covers the Sichuan Province with a grid resolution of 9 km × 9 km and Domain 3 covers Chengdu with a grid resolution of 3 km × 3 km. The input meteorological data used for the model were generated by Weather Research and Forecasting Model (WRF 3.9.1), according to the National Centers for Environmental Prediction Final (NCEP FNL). global tropospheric analysis business model dataset with a spatial resolution of 1° × 1° and a temporal resolution of 6 h. The Multi-resolution Emission Inventory of China (MEIC v1.3, 0.25° × 0.25°) of 2017 (http://www.meicmodel.org/) (accessed on 5 July 2022) [21,22] was used for the pollutant emissions inventory. The inventory contains 10 major air pollutants and CO_2_ emissions (NMVOC, NH_3_, PM_2.5_, PM_10_, CO, SO_2_, NO_x_, BC, OC, CO_2_) from five sectors: industry, power, residential, transportation and agriculture, with VOCs allocated according to the Carbon Bond-06 mechanism. MEIC is an emission inventory model framework based on bottom-up technology. The study used Inventory Spatial Allocate Tool (ISAT) to redistribute species on the temporal and spatial scales of Multi-resolution Emission Inventory of China of 2017 based on population and facilities, and established a high-resolution emission inventory (3 km × 3 km) of Chengdu, which was used as input to the CMAQ model. To eliminate the influence of initial conditions, the WRF started 7 days in advance.

### 2.2. Environmental Health Impact Assessment

The environmental population data, mortality data and socio-economic data were obtained from the 2017 Chengdu Statistical Bulletin of National Economic and Social Development. In 2017, Chengdu had a permanent population of 14.353 million, including 8.512 million urban and 5.841 million rural residents. The human health burden of short-term exposure to atmospheric pollutants can be calculated by the following formula [23,24]:

(1) $$M_{i}=\overset{n}{\underset{1}{\sum }}\frac{RR_{i}-1}{RR_{i}}\times Y_{0}$$

(2) $$RR_{i}=\exp\left(\beta \times \left(X-X_{0}\right)\right)$$

M_i_ is the total mortality caused by air pollutants exceeding a certain limit value, n is the number of days of environmental monitoring, Y_0_ is the baseline mortality, RR_i_ is the daily relative risk associated with short-term exposure to air pollutants, β is the empirical coefficient of concentration and health response obtained based on epidemiological investigation. In this study, the β% value is 0.38 (95%CI: 0.31–0.45) for every 10 μg·m^−3^ increase in PM_2.5_ concentration [25]. For every 10 μg·m^−3^ increase in O_3_ concentration, the β% value is 0.40 (95%CI: 0.30–0.50) [26]; X is the concentration of air pollutants. In this study, the daily average concentration of PM_2.5_ is adopted, and O_3_ is the maximum value of the eight-hour moving average concentration of O_3_ on the same day (MDA8 O_3_). X_0_ is the daily threshold concentration of air pollutants. The daily threshold concentration of PM_2.5_ is 15 μg·m^−3^, and the daily threshold concentration of O_3_ is 60 μg·m^−3^ according to the latest revised Global Air Quality Guidelines (AQG 2021) issued by the World Health Organization (WHO).

### 2.3. Economic Valuation of the Health Impacts

Statistical vital value (VSL) is commonly used to monetize the risk of premature death caused by air pollutants. This method mainly includes willingness to pay (WTP) and human capital (HC) methods [27]. Peng, et al. [28] investigated the average willingness to pay and vital statistical value of reducing air pollution health risks in Chengdu-Chongqing region in 2018 based on the single boundary dichotomic conditional value method, which is very consistent with this study. Economic losses from environmental health impacts are calculated as follows [29,30]:

(3) $$VSL_{2017}=VSL_{2018\;}\times \frac{I_{2017}}{I_{2018}}$$

(4) $$E_{i}=VSL_{2017}\times M_{i}$$

VSL is the vital statistical value of different years, and the vital statistical value of Chengdu in 2018 is 4.02 million yuan. I is the disposable income per capita of Chengdu in different years, which is 20,300 yuan in 2017 and 22,100 yuan in 2018. E_i_ is the economic loss caused by premature death.

## 3. Results

### 3.1. Model Validation

**Figure 2:**
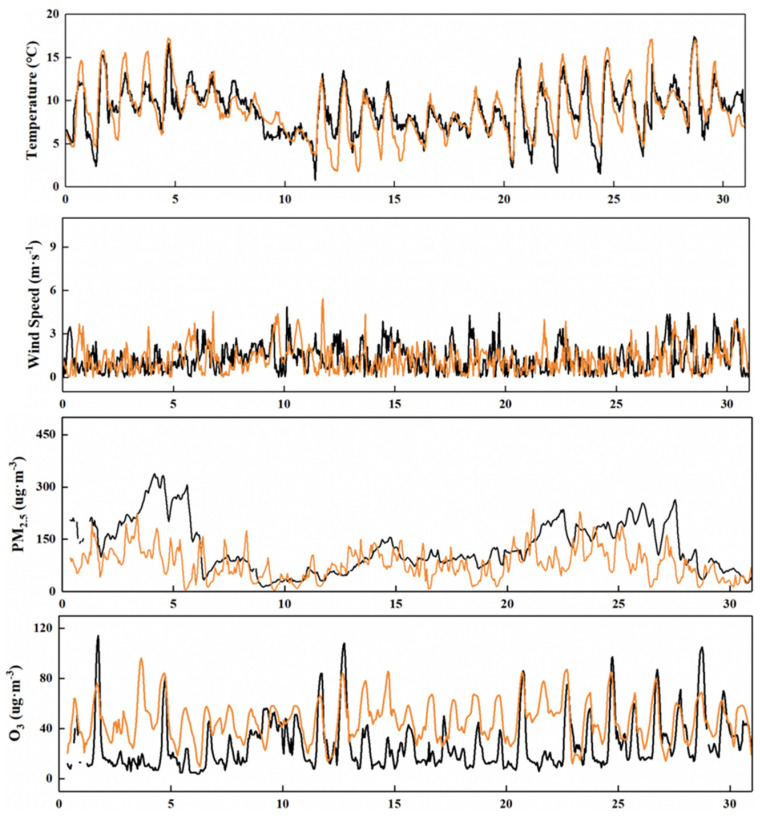
Time series of temperature at 2 m (T2), wind speed at 10 m (U10), PM_2.5_ and O_3_ concentration in Chengdu in January 2017. Black is the observed value and orange is the simulated value.

**Figure 3:**
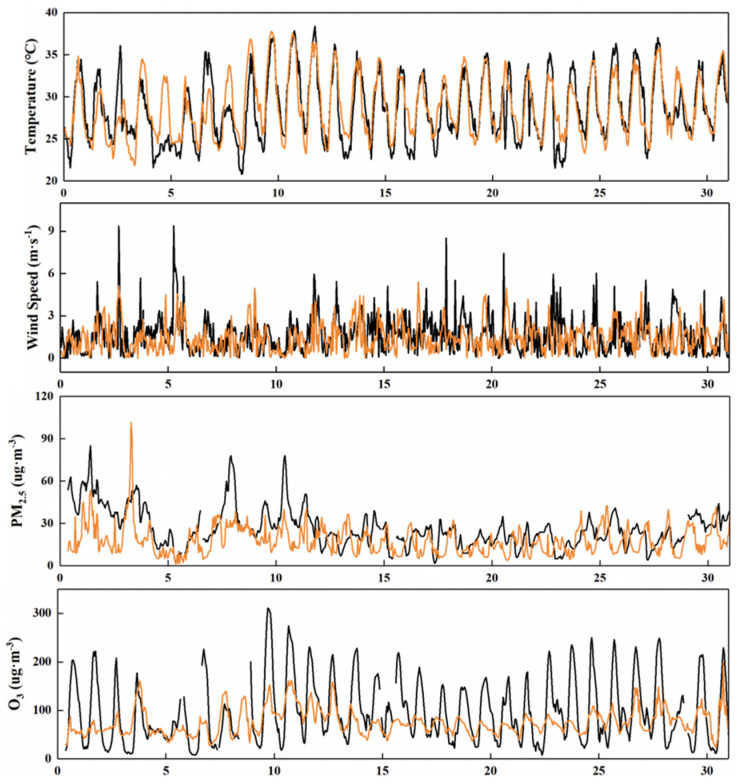
Time series of temperature at 2 m (T2), wind speed at 10 m (U10), PM_2.5_ and O_3_ concentration in Chengdu in July 2017. Black is the observed value and orange is the simulated value.

The simulation of meteorological conditions and pollution levels of key pollutants by the model was verified (Figure 2 and Figure 3). The temperature at 2 m (T2) provided by WRF simulation performed well in January and July, while the wind speed at 10 m (U10) performed poorly, which may be due to the unique basin topography of Sichuan and related to the parameterization scheme. However, the U10 simulation average can reach 87~98% of the observed value. The overall simulated variation in PM_2.5_ is relatively consistent, while O_3_ has a good grasp of the diurnal variation range. PM_2.5_ and O_3_ in Chengdu are in the low concentration range in summer and winter, respectively, and are underestimated, which is consistent with the study of Hu et al. [31]. The NMB of PM_2.5_ and O_3_ is about ±0.50 (Table 1 and Table 2), which also indicates that the change trend of pollutants is well understood [32]. The relevant meteorological parameters and pollutant data can be obtained from the model simulation to improve the monitoring data.

### 3.2. Spatial Distribution of Pollutant Emission

**Figure 4:**
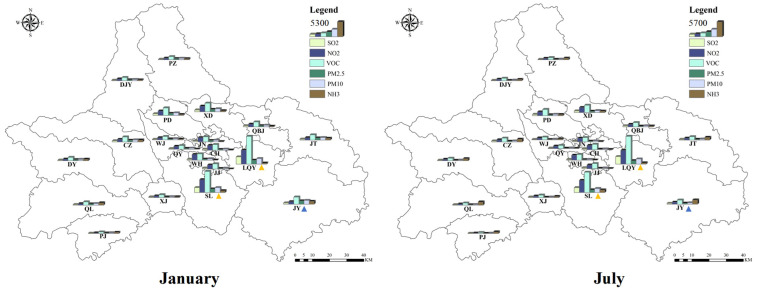
Emission distribution map of air pollutants in various regions of Chengdu (unit: t); the districts include Chenghua District (CH), Chongzhou City (CZ), Dayi County (DY), Dujiangyan (DJY), Jianyang City (JY), Jinniu District (JN), Jintang County (JT), Jinjiang District (JJ), Longquanyi District (LQY), Pengzhou City (PZ), Pidu District (PD), Pujiang County (PJ), Qingbaijiang District (QBJ), Qingyang District (QY), Qionglai City (QL), Shuangliu District (SL), Wenjiang District (WJ), Wuhou District (WH), Xindu District (XD), Xinjin District (XJ).

**Figure 5:**
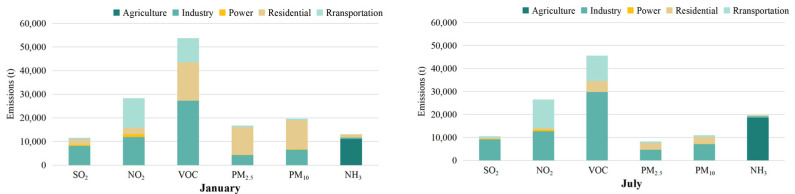
Emission of pollutants in agriculture, industry, power, residential and transportation sectors.

Based on the geographical and social information of Chengdu City, the multi-resolution Emission Inventory of China was used to make the Emission Inventory of Chengdu City and allocate its space. Figure 4 shows the emission distribution of air pollutants in various regions of Chengdu. Chengdu has jurisdiction over 12 municipal districts, three counties and five county-level cities, with a total area of 14,335 square kilometers. In January 2017, the emissions of SO_2_, NO_2_, NMVOC, PM_2.5_, PM_10_ and NH_3_ in Chengdu were 10619.96 t, 31023.33 t, 51228.96 t, 12666.22 t, 15369.05 t, 8625.33 t (Figure 5). In July, the pollutant emissions were 10314.87 t, 29867.42 t, 47378.47 t, 7401.54 t, 9992.24 t, 12804.24 t. Chengdu has a humid subtropical monsoon climate, heavy heating demand in winter and continuous industrial emissions have resulted in increased emissions of pollutants. Different from other pollutants, NH_3_ emissions in summer are much higher than in winter because NH_3_ mainly comes from animal husbandry and planting industry (Table 3). The emission of NH_3_ in the outer cities of Chengdu is relatively higher than that in the inner cities. Moreover, as a big agricultural country, China consumes high levels of nitrogen fertilizer and has large animal herds, which leads to vigorous industrial activities in summer [33,34].

Among the 21 districts in Chengdu, pollutant emissions are more noteworthy in Longquanyi District (LQY), Jianyang City (JY) and Shuangliu District (SL) (Table 3). In January 2017, LQY emitted SO_2_, NO_2_, NMVOC, PM_2.5_, PM_10_ of 2744.09 t, 5375.36 t, 10626.14 t, 1513.24 t, and 2119.62 t, respectively; SL emitted a total of 1869.86 t, 4947.65 t, 8092.08 t, 1412.34 t and 1874.20 t of SO_2_, NO_2_, NMVOC, PM_2.5_ and PM_10,_ respectively, and the PM_2.5_, PM_10_ and NH_3_ emissions in JY were 1333.38 t. In July 2017, the total emissions of SO_2_, NO_2_, NMVOC, PM_2.5_ and PM_10_ in LQY were 3054.91 t, 5624.78 t, 11328.73 t, 1425.80 t and 2080.96 t, respectively, the emissions of the SO_2_, NO_2_, NMVOC, PM_2.5_ and PM_10_ in SL were 1988.40 t, 4968.72 t, 8291.53 t, 1139.61 t and 1623.60 t, respectively, and the NH_3_ emissions in JY were 1793.86 T. LQY, JY and SL are located in the southeast area of Chengdu, and the area, total population and GDP of the three areas account for about 22.75%, 16.80% and 18.76% of Chengdu. The pollutants emitted from the three regions accounted for 26.51–52.27% of the total emissions in Chengdu, especially JY NH_3_ emissions accounted for 13.00–14.11% in Chengdu.

#### Source and Distribution of PM_2.5_

**Figure 6:**
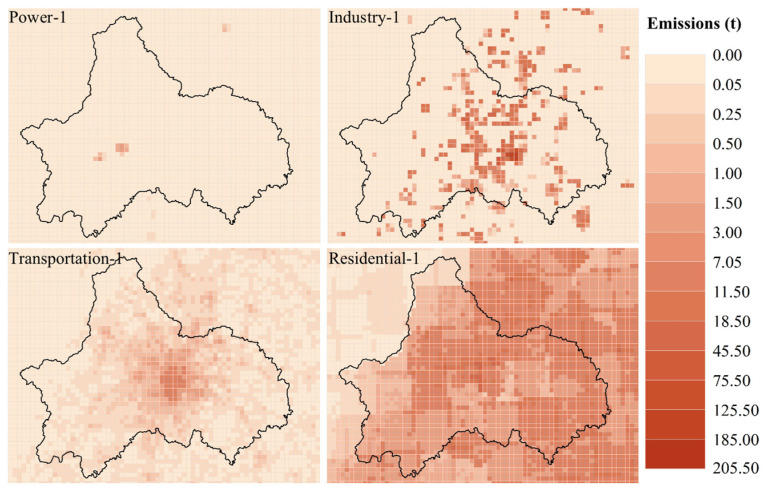
PM_2.5_ emission distribution of power, industry, residential and transportation sectors in Chengdu, January 2017.

**Figure 7:**
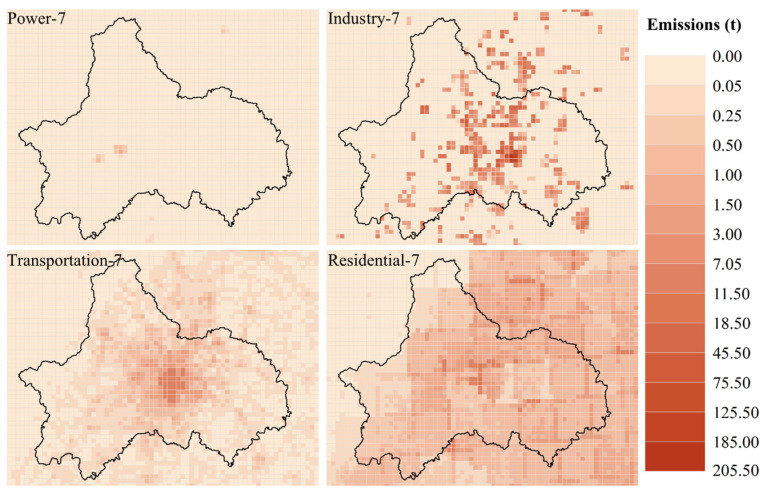
PM_2.5_ emission distribution of power, industry, residential and transportation sectors in Chengdu, July 2017.

**Figure 8:**
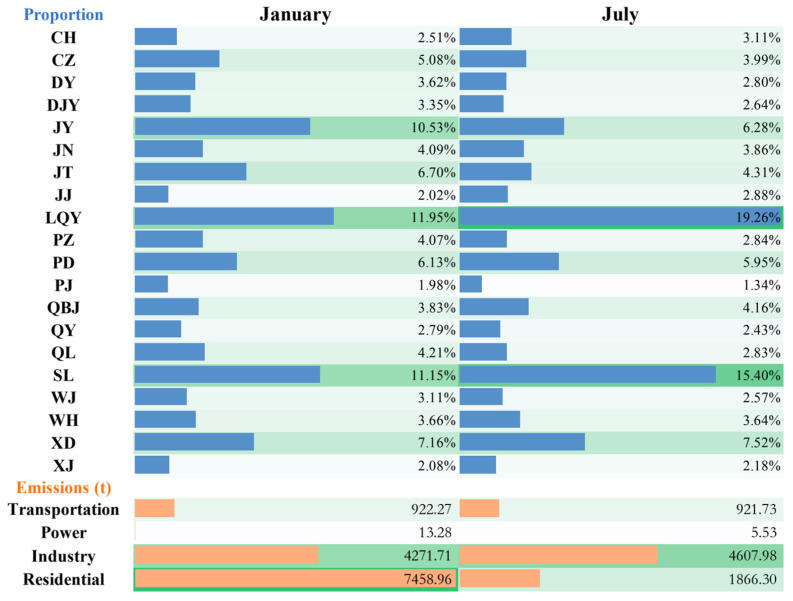
The proportion of PM_2.5_ emissions in Chengdu and the emissions by sectors; blue represents the ranking of PM_2.5_ emissions, orange represents the ranking of PM_2.5_ emissions by sector, and green is the background color changing with the ranking.

Figure 6 and Figure 7 show the distribution of PM_2.5_ emissions in power, industry, residential and transportation sectors in Chengdu in January and July 2017. In Chengdu, the emission of PM_2.5_ in January was higher than that in July in four sectors, but the emission of PM_2.5_ in power sector was not obvious, as reflected in the proportion of total PM_2.5_ emissions in January and July was lower than 1% of the total. The emissions from the transportation sector were consistent with the distribution of roads and the total PM_2.5_ emissions from the transportation sector accounted for 7.28% in January and 12.45% in July. The total PM_2.5_ emissions of industrial and residential sectors accounted for a large proportion, but showed different trends. The industrial sectors emissions were relatively stable in two seasons. In January, the total PM_2.5_ emissions of industrial sectors accounted for 33.72% of the total emissions, and in July, the total PM_2.5_ emissions of industrial sectors was 4271.71 t (Figure 8), which accounted for 62.26% of the total emissions. The PM_2.5_ emissions of the residential sector in winter are about four times that of the summer, with the total PM_2.5_ emissions of the residential sector in January 7458.96 t accounting for 58.88% of the total emissions, and in July 1866.29 t accounting for 25.21% of the total emissions. With the rapid economic development and the continuous improvement of the industrial system, the growth rate of emissions from industrial sources has slowed down. The urbanization process has caused the increase of emissions from urban residents and transportation sources with high population density. Although the reduction of solid fuel has improved this phenomenon, it should be paid attention to under the background of large base [35,36,37].

### 3.3. Variation in Pollutant Concentration

**Figure 9:**
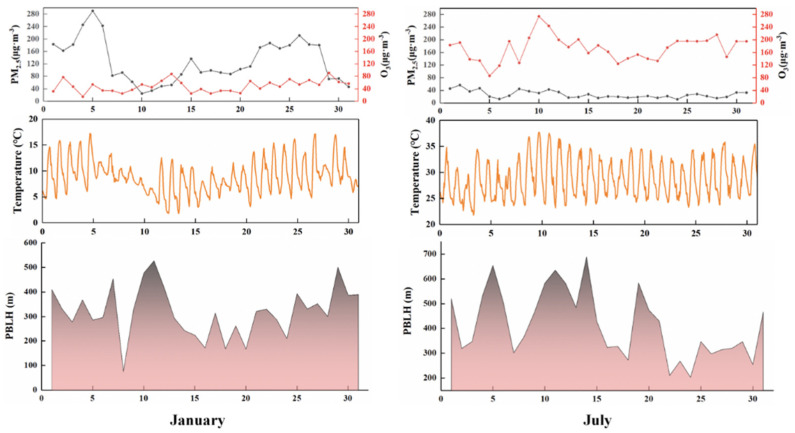
Daily changes in PM_2.5_, MDA8 O_3_, temperature, and PBLH.

In 2017, the PM_2.5_ concentration in Chengdu shows an opposite trend to O_3_ concentration (Figure 9). In winter, the demand and consumption of energy are large in various sectors, especially in the residential sector for household kitchens and heating. The PM_2.5_ concentration was high in winter and low in summer, with an average PM_2.5_ concentration of 128.79 ug·m^−3^ in January and 26.82 ug·m^−3^ in July. The concentration of O_3_ in winter was lower than that in summer. The average concentration of O_3_ in January was 27.42 ug·m^−3^, and it was 97.47 ug·m^−3^ in July. In January, PM_2.5_ concentration showed two large fluctuation peaks from 4th to 6th and from 21th to 28th, and in July, O_3_ concentration showed two large fluctuation peaks from 9th to 11th and from 25th to 28th. In 2017, the average temperature in January was 8.92 °C and the average temperature in July was 28.57 °C. Temperature can indirectly affect the generation and diffusion of pollutants by affecting the height of planetary boundary (PBLH) and the rate of chemical reactions. The variations in temperature and PBLH from January 1th to 6th and from January 21th to 28th are consistent, but the influence of such changes on the concentration of pollutants is not obvious. This may be because PM_2.5_ emissions are larger in winter and the overall PBLH height is low. In summer, high temperature and sunshine duration have a strong promoting effect on high O_3_ pollution events. On sunny days, under the strong ultraviolet radiation, VOCs and NO_x_ emitted by human being are easy to generate O_3_ through photochemical reactions. In July, Chengdu City had severe daily O_3_ pollution (265 ug·m^−3^~800 ug·m^−3^). The number of days with moderate pollution was (215 ug·m^−3^~265 ug·m^−3^) 2 days, and the number of days with mild pollution was (165 ug·m^−3^~215 ug·m^−3^) 16 days.

### 3.4. Health Burden

**Figure 10:**
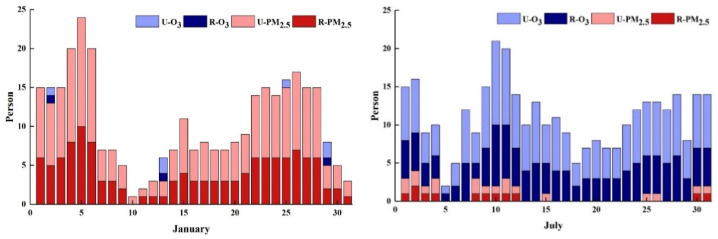
Health losses caused by PM_2.5_ and O_3_ in rural (R) and urban (U) areas of Chengdu in 2017.

As shown in Figure 10, the primary pollutant affecting human health in Chengdu in January 2017 was PM_2.5_, and it was O_3_ in July 2017. The industrial and residential sectors accounted for 92.61% of the total PM_2.5_ emissions in January. Therefore, only from the perspective of local emissions, the industrial and residential sectors contributed the most to the health loss caused by PM_2.5_, which is also consistent with the study of Hu, J.L. et al. [17]. PM_2.5_ pollution caused 351 deaths, O_3_ pollution caused 328 deaths, among which 276 deaths in rural areas and 413 deaths in urban areas were caused by pollutants. In January 2017, the mortality rate of PM_2.5_ pollution was 317 (95%CI: 247–388), including 129 (95%CI: 101–158) in rural areas and 188 (95%CI: 147–230) in urban areas. In July 2017, the mortality rate of PM_2.5_ pollution was 34 (95%CI: 22–46), including 14 (95%CI: 9–19) in rural areas and 20 (95%CI: 13–27) in urban areas. In January 2017, the mortality rate of O_3_ pollution was 10 (95%CI: 1–18), including 4 (95%CI: 1–8) in rural areas and 6 (95%CI: 1–11) in urban areas. In July 2017, the mortality rate of O_3_ pollution was 318 (95%CI: 22–46), including 129 (95%CI: 113–146) in rural areas and 189 (95%CI: 165–213) in urban areas.

### 3.5. Economic Losses

**Figure 11:**
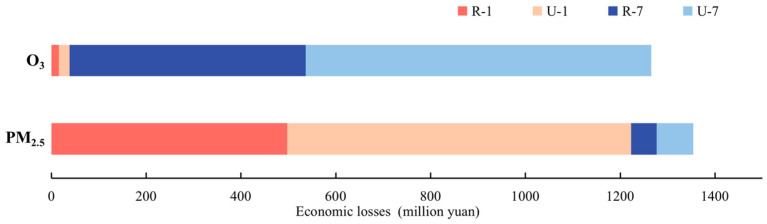
Economic losses caused by pollutants in urban (U) and rural (R).

The economic losses caused by PM_2.5_ and O_3_ air pollution were calculated based on vital statistical value (VSL). PM_2.5_ and O_3_ cause great economic losses in winter and summer, respectively (Figure 11). In January 2017, the health economic loss caused by PM_2.5_ in the air was 497.69 million yuan (95%CI: 389.65–609.55) in rural areas, and 725.29 million yuan (95%CI:567.11–887.32) in urban areas. In July 2017, the health economic loss caused by O_3_ in the air was 497.69 million yuan (95%CI: 435.94–563.26) in rural areas, and 729.15 million yuan (95%CI: 636.56–821.74) in urban areas. In January and July 2017, the health economic loss caused by PM_2.5_ was 1354.13 million yuan, accounting for 0.0974% of GDP of Chengdu in 2017. At the same time, the health economic loss caused by O_3_ was 1265.40 million yuan, accounting for 0.0910% of GDP of Chengdu in 2017.

## 4. Conclusions

The emission of pollutants in Chengdu is spatially focused on the area extending from downtown Chengdu to the southeast, and special attention should be paid to the LQY, JY and SL districts for regional emission control. The emission of PM_2.5_ pollutants from industrial and residential sectors is dominant. The seasonal fluctuation of PM_2.5_ emission from residential sectors is strong, and winter is much higher than summer.

The main pollutant is PM_2.5_ in winter and O_3_ in summer. Temperature can indirectly affect the previous diffusion of pollutants by affecting the boundary layer height, and high temperature has a promoting effect on the generation of O_3_. In Chengdu, PM_2.5_ and O_3_ pollution have a great impact on human health. In January and July, 351 deaths were caused by PM_2.5_ pollution, 328 deaths were caused by O_3_ pollution, 276 deaths in rural areas and 413 deaths in urban areas. The economic losses caused by PM_2.5_ and O_3_ accounted for a large proportion of the gross regional product (GDP) of Chengdu in 2017. In the future, attention should be paid to PM_2.5_ pollution control in winter and O_3_ pollution control in summer.

This study was based on the WRF-CMAQ model of pollutants and their health effects in Chengdu. Attention is still required to assess future research model parameterization schemes for the adjustment of the various regions and other pollutants to the health effects of towns and rural locations based on the same level as well as the external conditions, and the regional need to strengthen the monitoring of pollutants in rural areas at the same time, in order to fully understand the environmental pollution and environmental injustice caused by the urbanization process.

## Figures and Tables

**Table 1:** Model simulation in Chengdu, January 2017.

	NMB	NME	R
T2	0.006055	0.379440	0.781
U10	−0.009600	0.789565	0.048
PM_2.5_	−0.606118	0.746895	0.463
O_3_	0.409907	0.497384	0.457

**Table 2:** Model simulation in Chengdu, July 2017.

	NMB	NME	R
T2	0.017440	0.187526	0.799
U10	−0.139346	0.886220	−0.005
PM_2.5_	−0.495627	0.726415	0.400
O_3_	−0.293757	0.596831	0.577

**Table 3:** Pollutant emissions by region in Chengdu in January 2017 (units: t), July’s higher pollutant emissions than January are marked by blue numbers.

Area	SO_2_		NO_2_		VOC		PM_2.5_		PM_10_		NH_3_	
	January	July	January	July	January	July	January	July	January	July	January	July
CH	362.84	352.63	1837.83	1746.3	2142.11	**2173.64**	317.33	229.86	385.60	295.32	76.61	**93.79**
CZ	411.17	349.08	992.42	884.47	1882.10	1527.98	643.57	295.6	753.16	386.85	755.82	**1132.72**
DY	267.39	227.35	667.57	605.55	1298.13	1034.94	458.50	207.19	532.58	269.78	619.60	**898.18**
DJY	226.94	194.17	737.59	688	1267.57	1028.61	423.93	195.54	489.15	252.36	386.13	**577.88**
**JY**	485.79	349.07	998.13	842.46	2733.33	1689.08	1333.38	464.66	1469.92	561.1	1131.00	**1793.86**
JN	316.05	258.99	1627.17	1491.78	1936.17	1727.73	517.68	285.82	614.20	373.44	91.17	**96.89**
JT	340.29	257.07	919.50	821.11	1979.85	1362.59	848.87	319.1	943.85	390.39	720.80	**1115.05**
JJ	371.58	**378.77**	1593.35	1538.4	1991.42	**2079.72**	255.91	213.26	324.88	282.72	59.33	**70.72**
**LQY**	2744.09	**3054.91**	5375.36	**5624.78**	10628.14	**11328.73**	1513.24	1425.8	2119.62	2080.96	419.82	**538.8**
PZ	209.38	158.29	674.13	608.38	1232.13	877.41	515.10	210.53	583.39	266.34	461.81	**734.48**
PD	588.69	561.07	1695.12	1620.97	2858.39	2575.15	776.47	440.72	939.40	596.57	344.73	**525.08**
PJ	109.61	86.01	273.82	242.73	588.75	413.92	251.02	99.49	283.29	125.34	478.40	**702.09**
QBJ	409.09	407.01	1055.12	1029.7	1845.37	1716.33	484.60	307.96	607.43	430.99	241.07	**362.92**
QY	218.86	176.83	1146.26	1048.51	1420.53	1269.72	352.98	179.82	406.61	224.93	90.53	**111.48**
QL	227.57	175.44	584.84	515.94	1254.05	879.56	533.84	209.18	601.10	262.43	927.05	**1333.8**
**SL**	1869.86	**1988.4**	4947.65	**4968.72**	8092.08	**8291.53**	1412.34	1139.61	1874.20	1623.6	736.25	**1101.61**
WJ	215.41	185.35	869.13	820.68	1310.34	1106.12	393.76	189.87	454.47	243.25	292.75	**457.08**
WH	349.37	296.86	2202.87	2049.34	2431.32	2323.2	463.36	269.46	535.86	331.64	77.15	73.69
XD	668.57	636.46	2171.21	2085.58	3267.32	2972.39	907.06	556.5	1124.98	771.72	402.94	**616.03**
XJ	227.41	221.12	654.26	634.05	1069.88	1000.11	263.29	161.58	325.35	222.54	312.37	**468.08**

## Data Availability

Not applicable.
